# The relationship between lifestyle risk factors and depression in Korean older adults: a moderating effect of gender

**DOI:** 10.1186/s12877-021-02729-2

**Published:** 2022-01-05

**Authors:** Shinuk Kim

**Affiliations:** Department of Smart Information Communication Engineering, Sangmyung University, Cheonan, 330-720 South Korea

**Keywords:** Gender, Behavioral risk factors, Depression, Koreans, Nutritional inadequacy

## Abstract

**Background:**

Little is known regarding the role of gender as a possible modulator in determining the associations between lifestyle risk factors and depression in older adults.

**Objectives:**

This study examined whether gender modulates the relationship between depression and lifestyle risk factors in Korean adults aged 65 years and older (*n* = 3700).

**Methods:**

Data were obtained from the 2016 and 2018 Korea National Health and Examination Survey. The primary outcome was depression, assessed with the patient health questionnaire-9. As exposures, smoking habits, at-risk alcohol consumption, and physical inactivity were assessed with a standardized questionnaire. In addition, mean adequacy ratio (MAR) as an indicator of overall nutritional inadequacy was assessed with dietary intakes of macro- and micronutrients.

**Results:**

In men only, either two or three and more risk factors were significantly associated with higher depression risk (OR (95% confidence interval, CI) = 2.886 (1.003–8.299) and OR (95% CI) = 3.109 (1.064–9.097), respectively). In women only, either two or three and more risk factors were also significantly associated with higher depression risk (OR (95% CI) = 1.505 (1.067–2.124) and OR (95% CI) = 2.828 (1.527–5.239), respectively). In particular, the presence of smoking habits and MAR were the major determinants of depression (OR (95% CI) = 1.835 (1.09–3.10) and OR (95% CI) = 1.585 (1.125–2.233), respectively) in women only. Finally, a moderation analysis with the Hayes PROCESS Macro showed a significant moderating effect of gender (β (95% CI) = 0.633 (0.206 ~ 1.060)) on the relationship between risk factors and depression. In addition, the slope of the relationship was much steeper in women than in men.

**Conclusion:**

Current findings suggest that lifestyle risk factors are more closely associated with depression risk in women than in men.

## Introduction

Depression is a common mental disorder resulting in serious physical and mental health problems worldwide [1, 2]. In South Korea, the annual prevalence of major depressive disorder was 1.7, 2.5, and 3.1% in 2001, 2006, and 2011, respectively [3]. The prevalence is lower than in high-income countries (5.5%) and low-to-middle income countries (5.9%) but is rising steadily [4]. Furthermore, statistics in 2017 showed that South Korea has the highest suicide rate (at 23 deaths per 100,000 persons) among Organization for Economic Co-operation and Development countries [5] (https://data.oecd.org/healthstat/suicide-rates.htm). The total economic burden of depression was estimated to be 4049 million USD in 2005 [6]. By 2012, this figure had skyrocketed to 1.331 billion USD [7].

Etiologically, it is well established that depression is significantly associated with various lifestyle risk factors, including smoking [8], heavy alcohol consumption [9], physical inactivity [10], and unhealthy diet [11]. Depression is also associated with health conditions [12–14] and low socioeconomic status [15]. The relationships between lifestyle risk factors and the prevalence and incidence of depression have been also demonstrated in previous studies. For example, in a 7-year follow-up of the Komo-Ise cohort study, Tanaka et al. [16] showed that heavy alcohol consumption and physical inactivity were associated with a higher prevalence of depression in Japanese men, and obesity, inadequate sleep, and smoking were associated with a higher prevalence of depression in Japanese women. In an international cohort study of patients with muscle sclerosis, Taylor et al. [17] showed that healthy lifestyles, including moderate alcohol intake, being a non-smoker, diet quality, no meat or dairy intake, vitamin D supplementation, omega 3 supplement use, regular exercise, and meditation at baseline, were associated with lower incidences of depression.

Likewise, the findings from previous studies showed the relationships of depression with lifestyle risk factors in Korean populations. Interesting enough, women are more likely to suffer from depression [18, 19] and have a higher prevalence of depression than men [20]. Furthermore, an active or a passive healthy lifestyle compared to unhealthy lifestyle was associated with a lower risk of depressive symptoms in women, but such a relationship was not observed in men [21], implying that gender may play a role in determining the relationship between lifestyle risk factors and depression [22].

To the best of our knowledge, however, little is known regarding the moderating effect of gender on the relationships between lifestyle risk factors and depression in Korean populations. Therefore, we hypothesized that gender plays as a moderator in determining the relationship between modifiable lifestyle risk factors, including smoking, alcohol consumption, physical inactivity, and nutritional inadequacy, and depression risk in Korean geriatric populations. This study investigates whether gender moderates the association between lifestyle risk factors and depression in Korean older adults.

## Methods and materials

### Data source and study participants

**Fig. 1:**
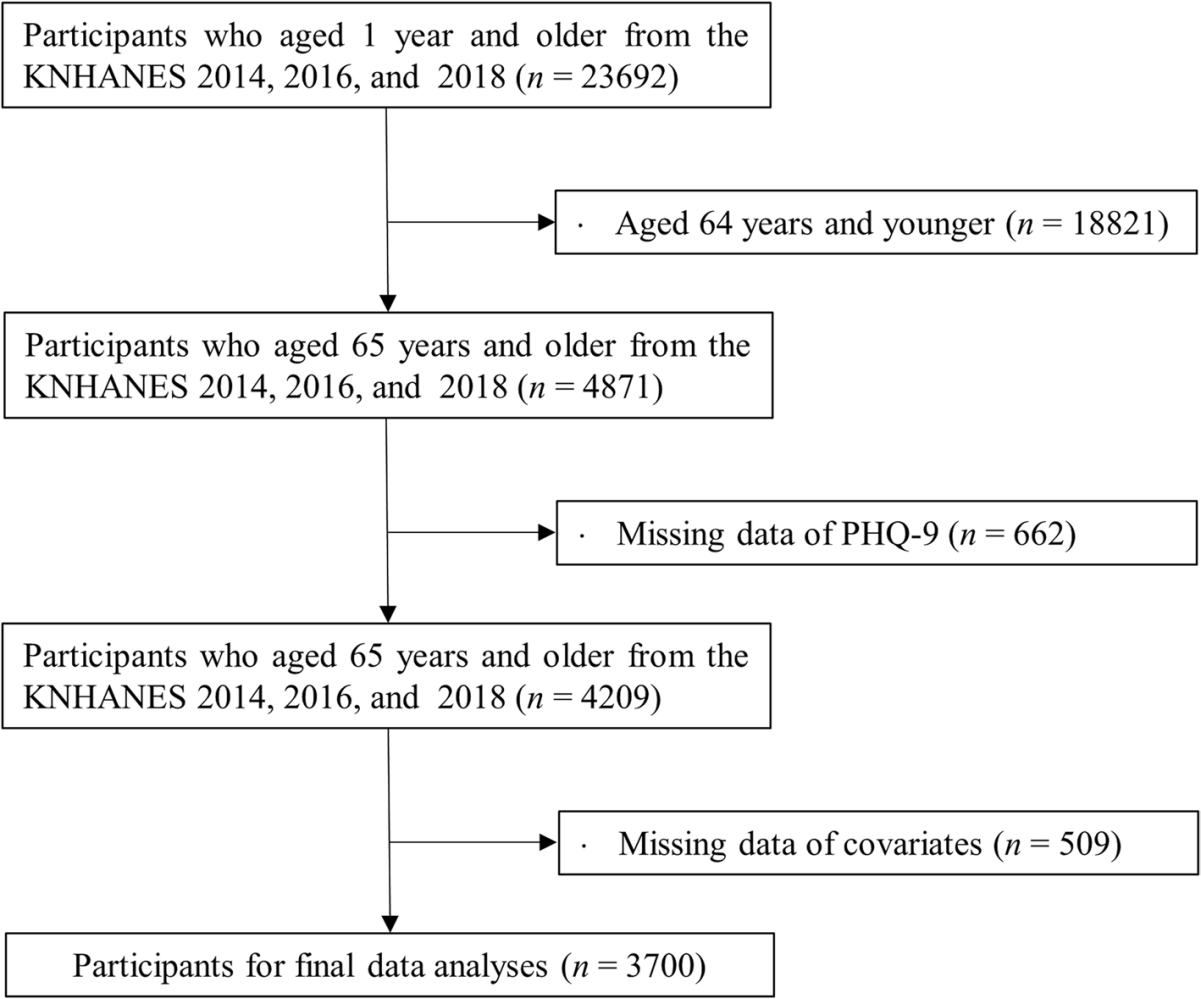
Flow chart of selection for study participants

The data used in the current study was obtained from the six and seven Korea National Health and Examination Survey (KNHANES) in 2041, 2016, and 2018, a nationwide survey designed to assess health and nutritional status in the Korean population. Initially, we selected all participants aged 1 year and older (*n* = 23,692) of the KNHANES VI and VII. We then excluded participants aged 64 years and younger (*n* = 18,821). Among the remaining 4871 participants, Additionally, we excluded those without PHQ-9 data (*n* = 662) and covariates and others (*n* = 509). Consequently, the 3700 participants aged 65 years and older who had all the parameters were included in final data analyses (Fig. 1). The Korea Centers for Disease Control and Prevention institutional review board reviewed and approved the KNHANES VII surveys (2018-01-03-P-A) in accordance with the Declaration of Helsinki. Informed consent forms were obtained from all the participants herein.

### Patient health Questionnaire-9 (PHQ-9)

The PHQ-9 is a self-reported version of the primary care evaluation of mental disorders (PRIME-MD) diagnostic instrument for major depressive disorders [23]. The Korean version of the PHQ-9 was downloaded from the PHQ website (https://www.phqscreeners.com/) and was used to match each of 9 items with criteria from the Diagnostic and Statistical Manual of Mental Disorders according to scores ranging from “0” (not at all) to “3” (nearly every day). Subjects were classified based on the severity of depressive symptoms (none, 0; minimal, 1–4; mild, 5–9; moderate, 10–14; moderately severe, 15–19; or severe, 20–27) [23]. In the current study, a PHQ-9 score of ≥10 was defined as having major depressive disorders (MDD). The cutoff value of 10 for screening MDD using the Korean version of PHQ-9 was previously tested and validated [24].

### Lifestyle risk factors

Lifestyle risk factors measured in the study, including current or past smoking, at-risk alcohol consumption, physical inactivity, and overall nutritional inadequacy, are modifiable behaviors and exposures that can raise or lower a person’s risk of diseases [25]. In addition, those lifestyle risk factors are the key components of the Healthy Promotion Programs by the Korea Ministry of Health and Welfare (https://www.khealth.or.kr/) and associated with depression and/or depressive symptoms in Korean populations [26–29].

With regard to smoking, respondents were classified as never smokers and past or current smokers. At-risk alcohol intake was defined as having seven or more (five or more for women) glasses of alcohol per occasion per two or more times per week [30]. Physical inactivity was defined as not participating in at least 150 min of moderate physical activity (PA) per week or 75 min of vigorous PA or a combination of moderate and vigorous PA (https://www.who.int/news-room/fact-sheets/detail/physical-activity).

With respect to overall nutritional inadequacy, dietary intake of macronutrients (i.e., carbohydrates, fats, and proteins) and micronutrients (i.e., vitamins A and C, thiamine, riboflavin, niacin, phosphorous, calcium, and iron) was assessed with a 24-h (h) recall method. Trained interviewers conducted computer-assisted personal interviews to assess all food items ingested during the previous 24 h. Mean adequacy ratio (MAR), which represents a population’s overall nutritional adequacy, was then calculated by using the following equation: MAR = (sum of NAR / number of nutrients) × 100. The nutrient adequacy ratio (NAR) represents an individual’s intake of a nutrient as a percentage of the age- and gender-specific recommended dietary allowance (RDA) for that nutrient [31] accessed on 24 May 2021. For respondents, MAR was dichotomized as adequacy (100% and higher) or inadequacy (less than 100%) based on the age- and gender-specific RDA for macro- and micronutrients. A score of 1 was given for each lifestyle risk factors. Number of lifestyle risk factors was defined as a total score of the risk factors.

### Covariates

The covariates used in this study were age (continuous), gender (categorical: male or female), body mass index (quantitative), education (categorical: elementary school or lower, middle or high school, university or higher), income (quantitative), and marital status (categorical: yes or no). Body mass index (BMI) was calculated as weight divided by height squared (kg/m^2^).

### Statistics

Normality of data distribution was confirmed with quantile-quantile (Q-Q) plotting, and the absence of multicollinearity was assessed by a variance of inflation factor (VIF). Descriptive statistics between men and women in measured parameters were performed with Student’s t-tests and chi-squared tests for continuous and categorical variables, respectively, and they are presented as mean ± standard deviations (SDs) and numbers (n) and percentages (%), respectively.

Multivariate logistic regression was performed to estimate odds ratios (ORs) and 95% confidence intervals (CI) of MDD with a PHQ-9 score of > 10 (categorical) by lifestyle risk factors-based subgroups (categorical) before and after adjustments for the covariates. For the classification of lifestyle risk factors, 0 and 1 risk factors were collapsed and classified as < 1 risk factor because of a small proportion of 0 risk factor (2.4% in total/1.9% in men and 2.9% in women). Likewise, 3 and 4 risk factors were collapsed and classified as > 3 risk factors because the proportion of 4 risk factors was 1.7% in total (3.6% in men and 0.2% in women). Consequently, lifestyle risk factors-based subgroups were classified as < 1 risk factor or 2 risk factors or > 3 risk factors.

**Fig. 2:**
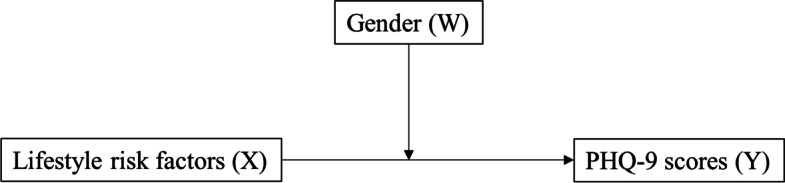
A hypothetical model of moderation analysis

Finally, we examined the moderating effect (or the interaction effect) of gender (moderator, W) on the relationship between number of lifestyle risk factors (continuous, X) and PHQ-9 score (continuous, Y), as shown in Fig. 2, with the Hayes PROCESS Macro using ordinary least squares (OLS) regression. For moderation analysis, gender was dummy coded (i.e., 0 = male and 1 = female), and lifestyle risk factors and PHQ-9 scores were treated as continuous variables. After centering number of lifestyle risk factors and PHQ-9 score and computing the interaction (i.e., X × W) term [32], the predictor and the interaction were entered into a simultaneous regression model. Age, alcohol consumption, sleeping time, body mass index, income, education level, marital status, residence area, and comorbidities were controlled in the model. The model was also tested with bias-corrected bootstrapping (*n* = 5000) and 95% confidence intervals (CI). If a 95% bootstrapped CI does not include zero, it indicates the relationship is statistically significant. All other statistical significances were tested at *p* = 0.05 by using statistical software PASW SPSS WIN 27.0 (SPSS Inc., Chicago, IL).

## Results

Table 1 presents the descriptive statistics of the study participants. Men had lower BMI (*p* <  0.001) but had higher incomes (*p* <  0.001), higher education levels (*p* <  0.001), and higher rates of past/current smoking (*p* <  0.001), at-risk alcohol consumption (*p* <  0.001), and physical inactivity (*p* <  0.001) than women. Men also had lower rates of overall nutritional inadequacy (*p* < 0.001), lower PHQ-9 scores (*p* < 0.001), lower prevalence of depression (*p* < 0.001), and lower numbers of comorbidities (*p* < 0.001) than women.

**Table 1:** Descriptive statistics of study participants

Variable	Men (*n* = 1622)	Women (*n* = 2078)	Total (*n* = 3700)	*p* value
Age (years), mean (SD)	72.5 (5.0)	72.5 (5.0)	72.6 (5.0)	0.356
Body mass index (kg/m^2^), mean (SD)	23.7 (2.9)	24.5 (3.3)	24.1 (3.1)	< 0.001
Income (10,000 won/month), mean (SD)	243 (258)	202 (239)	220 (249)	< 0.001
Education, n (%)				< 0.001
Lower than elementary	515 (31.8)	1411 (67.9)	1926 (52.1)	
Middle/high	787 (45.8)	556 (26.8)	1343 (36.3)	
College or higher	320 (19.7)	111 (5.3)	431 (11.6)	
Marital status, n (%)				0.788
Unmarried	12 (0.7)	18 (0.8)	29 (0.8)	
Married	1610 (99.3)	2061 (99.2)	3671 (99,2)	
Past/current smoking, n (%)	1279 (78.9)	108 (5.2)	1387 (37.5)	< 0.001
At-risk alcohol consumption, n (%)	177 (10.9)	34 (1.6)	211 (5.7)	< 0.001
Physical inactivity, n (%)	1404 (41.7)	1963 (58.3)	3367 (91.0)	< 0.001
Nutritional inadequacy, n (%)	803 (49.5)	1359 (65.4)	2162 (58.4)	< 0.001
**Dietary intakes**				
Caloric intake (kcal/day), mean (SD)	1956.7 (733.4)	1465.2 (587.9)	1680.6 (699.5)	< 0.001
CHO intake (g/day), mean (SD)	328.1 (124.9)	266.2 (108.9)	293.3 (120.2)	< 0.001
Fat intake (g/day), mean (SD)	31.2 (24.4)	22.4 (18.3)	26.3 (21.7)	< 0.001
Protein (g/day), mean (SD)	65.0 (31.8)	47.1 (24.1)	54.9 (29.2)	< 0.001
Vitamin A (μgRAE/day), mean (SD)	340.5 (329.2)	268.6 (257.5)	299.8 (292.9)	< 0.001
Vitamin C (mg/day), mean (SD)	74.4 (84.9)	65.7 (76.2)	69.5 (80.2)	0.001
Thiamine (mg/day), mean (SD)	1.5 (0.8)	1.2 (0.6)	1.3 (0.7)	< 0.001
Riboflavin (mg/day), mean (SD)	1.3 (0.8)	1.0 (0.6)	1.1 (0.7)	< 0.001
Niacin (mg/day), mean (SD)	13.2 (7.0)	9.5 (5.4)	11.1 (6.4)	< 0.001
Phosphorus (mg/day), mean (SD)	1049.8 (470.0)	786.0 (378.9)	901.7 (441.1)	< 0.001
Calcium (mg/day), mean (SD)	498.6 (341.6)	384.0 (276.8)	434.2 (312.1)	< 0.001
Iron (mg/day), mean (SD)	14.7 (10.3)	11.3 (9.1)	12.8 (9.8)	< 0.001
PHQ-9 score, mean (SD)	2.0 (3.5)	3.4 (4.6)	2.8 (4.2)	< 0.001
Depression, n (%)	68 (4.2)	209 (10.1)	277 (7.5)	< 0.001
Number of comorbidities, n (%)				< .0001
0	380 (23.4)	268 (12.9)	648 (17.5)	
1	529 (32.6)	564 (27.1)	1093 (29.5)	
> 2	713 (44.0)	1246 (60.0)	1959 (52.9)	

*PHQ* Patient Health Questionnaire

Table 2 presents the associations between individual lifestyle risk factors and depression. In total, smoking was associated with greater odds of depression (OR (95% CI) = 1.531 (0.986 ∼ 2.316)). In addition, overall nutritional inadequacy was also associated with greater odds of depression (OR (95% CI) = 1.559 (1.170–2.078)) even after adjustments for age, gender, BMI, income, education, marital status, residence area, and number of comorbidities. Gender-specific analysis showed that smoking was significantly associated with greater odds of depression among women (OR (95% CI) = 1.835 (1.09–3.10)) but not among men (OR (95%CI) = 1.14 (0.60–2.17)). Likewise, overall nutritional inadequacy was significantly associated with greater odds of depression among women (OR (95% CI) = 1.585 (1.125–2.233)) but not among men (OR (95% CI) = 1.518 (0.897–2.570)).

**Table 2:** Odds ratios (ORs) and 95% confidence intervals (CIs) of depression according to individual lifestyle risk factors

Predictors	Model 1		Model 2	
	OR (95% CI)	*p* value	OR (95% CI)	*p* value
Total				
Smoking	0.597 (0.454–0.786)	< 0.001	1.531 (0.986–2.316)	0.050
At-risk alcohol intake	0.374 (0.404–1.331)	0.308	1.151 (0.617–2.147)	0.657
Physical inactivity	1.789 (1.050–3.048)	0.032	1.194 (0.687–2.074)	0.529
Nutritional inadequacy	2.010 (1.529–2.643)	0.001	1.559 (1.170–2.078)	0.002
Men only-				
Smoking	1.263 (0.669–2.384)	0.471	1.141 (0.599–2.172)	0.688
At-risk alcohol intake	1.258 (0.613–2.584)	0.531	1.453 (0.693–3.049)	0.323
Physical inactivity	2.555 (0.921–7.089)	0.072	1.851 (0.652–5.252)	0.247
Nutritional inadequacy	1.920 (1.156–3.189)	0.012	1.518 (0.897–2.570)	0.120
Women only				
Smoking	2.000 (1.192–3.355)	0.009	1.835 (1.085–3.104)	0.024
At-risk alcohol intake	0.863 (0.262–2.849)	0.810	0.775 (0.232–2.593)	0.679
Physical inactivity	1.061 (0.560–2.009)	0.857	0.933 (0.483–1.802)	0.837
Nutritional inadequacy	1.723 (1.239–2.396)	0.001	1.585 (1.125–2.233)	0.009

Model 1: unadjusted

Model 2: adjusted for age (and gender for total), body mass index, income, education level, marital status, residence area, and number of comorbidities

Table 3 represents depression risk stratified by number of lifestyle risk factors. In total, two risk factors and three and more risk factors were significantly associated with increased depression risk (OR (95% CI) = 1.786 (1.308–2.440) and OR (95% CI) = 2.979 (1.903–4.632), respectively) in comparison to one or zero risk factor (OR = 1). The increased ORs for two risk factors and three and more risk factors remained statistically significant (OR (95% CI) = 1.620 (1.174–2.236) and OR (95% CI) = 2.341(1.473–3.719), respectively) even after adjustments for age, gender, BMI, income, education, marital status, residence area, and number of comorbidities. In men only, two risk factors and three and more risk factors were significantly associated with increased depression risk (OR (95% CI) = 3.446 (1.208–9.829) and OR (95% CI) = 4.510 (1.580–12.875), respectively) in comparison to one or zero risk factor (OR = 1). The increased ORs for two risk factors and three and more risk factors remained statistically significant (OR (95% CI) = 2.886 (1.003–8.299) and OR (95% CI) = 3.109 (1.064–9.097), respectively) even after adjustments for age, BMI, income, education, marital status, residence area, and number of comorbidities. In women only, two risk factors and three and more risk factors were significantly associated with increased depression risk (OR (95% CI) = 1.634 (1.174–2.273) and OR (95% CI) = 3.363 (1.842–6.139), respectively) in comparison to one or zero risk factor (OR = 1). The increased ORs for two risk factors and three and more risk factors remained statistically significant (OR (95% CI) = 1.505 (1.067–2.124) and OR (95% CI) = 2.828 (1.527–5.239), respectively) even after adjustments for all the covariates.

**Table 3:** Odds ratios (ORs) and 95% confidence intervals (CIs) of depression according to lifestyle risk factors-based subgroups

Predictors	Model 1		Model 2	
	OR (95% CI)	*p* value	OR (95% CI)	*p* value
Classification of lifestyle risk factors (total)				
< 1	1 (ref)		1 (ref)	
2	1.786 (1.308–2.440)	< 0.001	1.620 (1.174–2.236)	0.003
> 3	2.979 (1.903–4.632)	< 0.001	2.341 (1.473–3.719)	0.001
Classification of lifestyle risk factors (men only)				
< 1	1 (ref)		1 (ref)	
2	3.446 (1.208–9.829)	0.021	2.886 (1.003–8.299)	0.049
> 3	4.510 (1.580–12.875)	0.005	3.109 (1.064–9.097)	0.038
Classification of lifestyle risk factors (women only)				
< 1	1 (ref)		1 (ref)	
2	1.634 (1.174–2.273)	0.004	1.505 (1.067–2.124)	0.020
> 3	3.363 (1.842–6.139)	< 0.001	2.828 (1.527–5.239)	0.001

Model 1: unadjusted

Model 2: adjusted for age (and gender for total), body mass index, income, education level, marital status, residence area, and number of comorbidities. Lifestyle risk factors include smoking, at-risk alcohol consumption, physical inactivity, and overall nutritional inadequacy

**Fig. 3:**
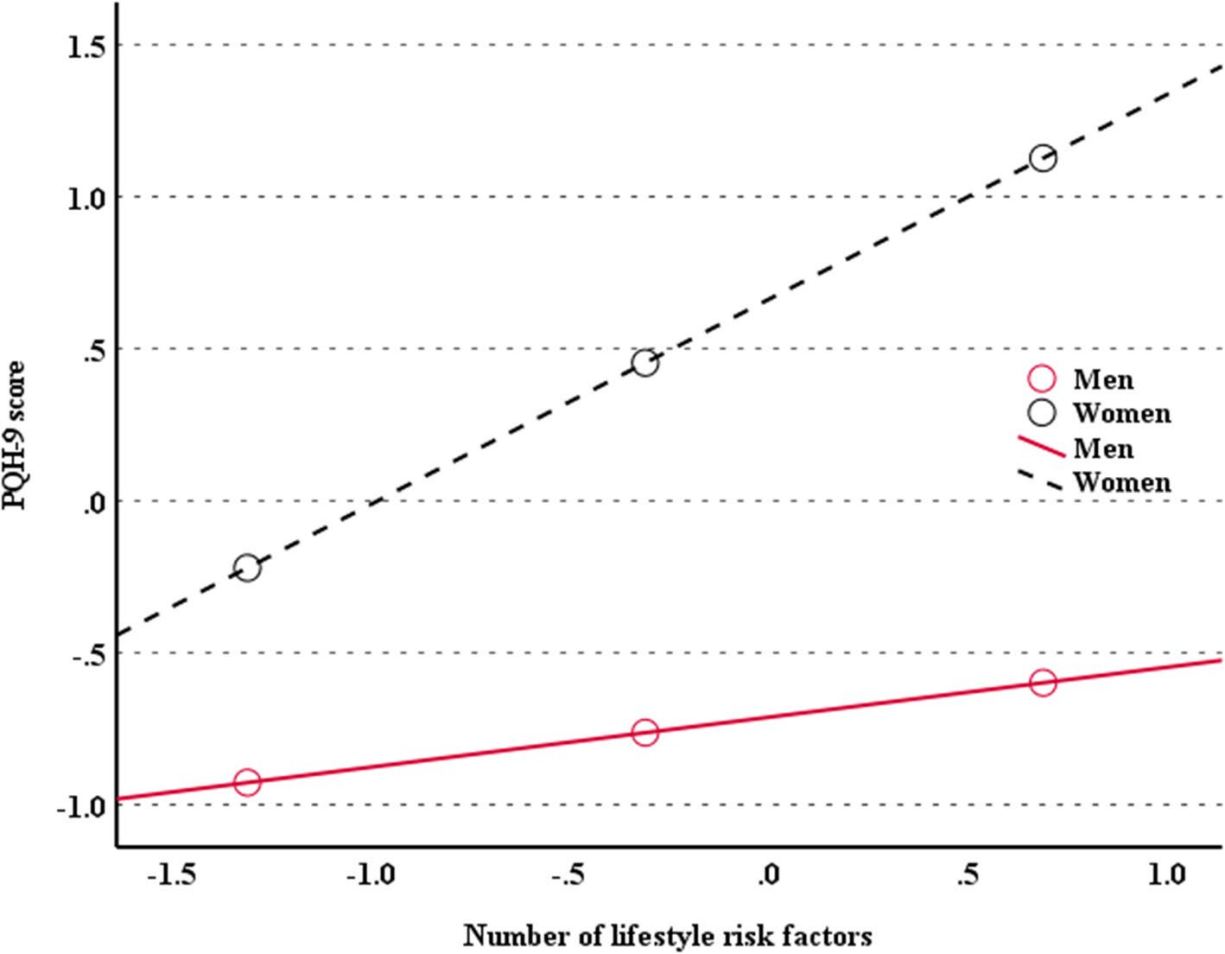
The moderating effect of gender in the relationship between lifestyle risk factors (i.e., smoking, physical inactivity, and overall nutritional inadequacy) depression risk in Korean older adults

Table 4 represents the moderating analysis of gender for the relationship between depression and lifestyle risk factors. There was a significant interaction between gender and risk factors on depression (β (95% CI) = 0.480 (0.173 ~ 0.787)). The moderating effect of gender remained significant (β (95% CI) = 0.509 (0.204 ~ 0.814)) even after adjustments for the covariates. Since the interaction term (X × W) in the model was significant, we wanted to probe the interaction to better interpret the moderating effect of gender on the relationship between lifestyle risk factors and PHQ-9 score. The slope of PHQ-9 score on lifestyle risk factors was much steeper in female gender than in male gender, as depicted in Fig. 3.

**Table 4:** Relationship of PHQ-9 score with lifestyle risk factors: a moderating effect of gender

Predictors	Unstandardized coefficients	SE	t	*p*	95% CI	
					Lower	Upper
Model 1 (*R*^2^ = 0.039, F_(3, 3696)_ = 49.958, *p* < 0.001)						
LRF	−0.218	0.245	−0.889	0.374	−0.697	0.262
Gender	1.616	0.143	11.32	< 0.001	1.336	1.896
Interaction	0.480	0.157	3.067	0.002	0.173	0.787
Model 2 (*R*^2^ = 0.062, F_(9, 3690)_ = 24.372, *p* < 0.001)						
LRF	−0.345	0244	−1.417	0.157	−0.822	1.133
Gender	1.373	0.165	8.781	< 0.001	1.067	1.680
Interaction	0.509	0.156	3.272	0.011	0.204	0.814

*LRF* Lifestyle risk factors, *PHQ* Patient healthcare question

Model 1: unadjusted

Model 2: adjusted for age, alcohol consumption, sleeping time, body mass index, income, education level, marital status, residence area, and comorbidities

Unstandardized coefficients represent the slope of the line between the predictor variable (i.e., lifestyle risk factors, gender, and lifestyle risk factors by gender) and the dependent variable (PHQ-9 score)

## Discussion

This cross-sectional and population-based study examines gender differences in the relationships between lifestyle risk factors (i.e., smoking habits, at-risk alcohol consumption, physical inactivity, and overall nutritional inadequacy) and depression in Korean older adults. Results show that regardless of gender, accumulation of lifestyle risk factors is significantly associated with depression. In addition, smoking and overall nutritional inadequacy are the major determinants of depression risk in women only. Novel to this study is that the slope of the relationship between the four risk factors and depression is much steeper in women than in men, implying that women might be more vulnerable to depression associated with lifestyle risk factors.

The findings of the current study are in line with findings from previous studies. For example, in a population-based study involving 3300 Chinese adults (1973 women) aged 35–74 years, Cui et al. [33] examined the associations between health conditions, lifestyle factors, and depression. That study finds that diabetes (both pre-diabetes and previously diagnosed diabetes) and physical activity were independent determinants of depression among male participants, while waist circumference was an independent determinant of depression among female participants. In a study comprised of 665 Japanese adults (347 women) aged 65 years and older, Katsumata et al. [34] examined the relative contributions of four risk domains including demographic characteristics, health and disability, stress, and social networks for depression and found that stress was the major risk domain for men, while health and disability status were the major risk domains for women. Chang et al. [35] examined the relationships between lifestyle risk factors and depressive symptoms in 1020 Taiwanese community-dwelling older adults and found that regular exercise and the regular consumption of vegetables were associated with a lower risk of depressive symptoms in men, while regular exercise was significantly associated with low depressive symptoms in women.

Gender differences in depression and/or depressive symptoms have been found in Korean populations. For example, Jang et al. [36] examined gender differences in depressive symptoms in a sample of 230 older Korean-American immigrants and found a significant interaction between gender and chronic conditions on depressive symptoms. Specifically, women with chronic conditions had a higher prevalence of depressive symptoms. Roh et al. [37] examined the relationship of depression and cognitive impairment with the risk of falling in 7150 Korean older adults (4147 women) who submitted complete responses to a three-year follow-up in the Living Profiles of Older People Survey in Korea, finding that depression was more closely associated with the risk of falling in men than in women. In a population-based study involving 7554 Korean adults aged 45–74 years (60% women), Lee et al. [38] examined the relationship between depressive symptoms and carotid atherosclerosis. That research shows that women with depressive symptoms were more vulnerable to the risk of carotid atherosclerosis than women without depressive symptoms, with no such relationship observed in men. By conducting a secondary analysis of data (1938 men and 2404 women aged 40 years) obtained from the 2010–2014 Korean National Health and Nutrition Examination Survey, Ra and Kim [39] showed that depression was significantly associated with greater waist circumference and higher triglycerides in women but not in men. By analyzing data (*n* = 5103) obtained from the seventh Korea National Health and Nutrition Examination Survey 2018, Lee and Kim [40] showed that dietary habits such as skipping lunch, practicing a meal frequency of two times per day, and practicing a lunch frequency of 3-4times per week were significant predictors of depression risk in women. These findings imply that unhealthy dietary patterns are a potential contributor to gender differences in the relationship between lifestyle risk factors and depression. Considering the cross-sectional nature of previous studies including the current one, however, gender difference in the relationship should be further tested and confirmed in a cause-and-effect manner in a well-designed longitudinal cohort study.

Several explanations can be given for the gender differences observed in the current study. First, the high odds of women to depression associated with lifestyle risk factors may reflect health consequences of the loss or reduction of antidepressant effects of estrogen due to menopause [41], female-specific reproductive events (i.e., perimenstrual changes, pregnancy, postpartum periods, and menopause [42], and women-specific risk variants in the genetic architecture of depression [43]. Second, unhealthy eating behaviors in women may lead to overall nutritional inadequacy, potentially contributing to increased depression risk [44]. However, a bidirectional relationship between dietary behaviors and depression is also possible; depressed persons are likely to have unhealthy dietary patterns, resulting in undernutrition and/or malnutrition [45]. Caution is necessary in interpreting directionality in the relationship between overall nutritional inadequacy and depression observed herein. Third, health inequity associated with low socioeconomic status (SES) and demographic factors may also contribute to the higher susceptibility of women to chronic illness and to mental illness [15]. Lastly, Smoking may play as another mediator in determining the relationship between lifestyle risk factors and depression. By analyzing data from the participants aged 91 years and older (*n* = 31,814) of the 2008–2012 Korean National Health and Nutrition Examination Survey (KNHANES), Kim et al. [46] showed that current female smokers were more likely to have a depressive episode, suicidal ideation and attempts, and psychiatric counselling compared to current male smokers. Consequently, it cannot be ruled out the possibility that gender difference in the relationship may reflect gender-specific smoking rate and its relationship with mental health. Taken together, although the exact mechanisms underlying the gender differences observed in the current study are unclear, it is likely that the abovementioned factors (either singularly or accumulatively) may be intertwined.

This study has some limitations. First, although the PHQ-9 score-based assessment of MDD was previously tested and validated in Korean elderly populations [24], the instrument is a subjective screening test and not cover all depression criteria, especially functional impairment. Therefore, an additional item of assessing depression-specific functional impairments should be supplemented so that making type II error or false negative can be minimized in a future study. Second, the cross-sectional nature of the study limits any cause-and-effect explanation for the current findings. Third, a bidirectional relationship between depression and lifestyle risk factors seems possible. That is, accumulation of lifestyle risk factors may contribute to increased depression risk, and vice versa, and this remains to be further investigated in a future study. Fourth, it is possible that the modulating effects of gender on the relationship between lifestyle risk factors and depression differ by certain confounders such as age and socioeconomic status, and this remains to be further addressed in a future research. Lastly, the data used in the current study was obtained on the basis of binary gender categories so that we could not address any non-binary gender effect(s) on the study outcomes.

Despite limitations, this study also has strengths. First, it is a population-based study with a relatively high response rate and large sample size. Second, to the best of our knowledge, this study is the first to report that lifestyle risk factors may play a role for gender difference in the prevalence of depression.

## Conclusion

This population-based study finds that 1) lifestyle risk factors, especially smoking and overall nutritional inadequacy, are significantly associated with depression risk in women but not in men and 2) the slope of the relationship between the risk factors and depression is much steeper in women than in men, implying the importance of implementing gender-specific interventions for at-risk populations.

## Data Availability

The datasets used and/or analysed during the current study are available from the corresponding author on reasonable request.
